# Edema Resolution and Clinical Assessment in Poor-Grade Subarachnoid Hemorrhage: Useful Indicators to Predict Delayed Cerebral Infarctions?

**DOI:** 10.3390/jcm10020321

**Published:** 2021-01-17

**Authors:** Ramon Torné, Jhon Hoyos, Laura Llull, Ana Rodríguez-Hernández, Guido Muñoz, Ricard Mellado-Artigas, Daniel Santana, Leire Pedrosa, Alberto Di Somma, Luis San Roman, Sergio Amaro, Joaquim Enseñat

**Affiliations:** 1 Neurological Surgery Department, Hospital Clinic of Barcelona, 08036 Barcelona, Spain; adisomma@clinic.cat (A.D.S.); jensenat@clinic.cat (J.E.); 2 Hospital Clinic, University of Barcelona and Institut d’Investigació Biomèdica August Pi i Sunyer (IDIBAPS), 08036 Barcelona, Spain; blllull@clinic.cat (L.L.); lepedrosa@clinic.cat (L.P.); samaro@clinic.cat (S.A.); 3 Comprehensive Stroke Unit, Neurology Department, Hospital Clinic of Barcelona, 08036 Barcelona, Spain; santana@clinic.cat; 4 Neurological Surgery Department, Germans Trías i Pujol Hospital, 08916 Badalona, Spain; ana.neurosurgery@hotmail.com; 5 Intensive Care Unit, Hospital Clinic of Barcelona, 08036 Barcelona, Spain; gamunoz@clinic.cat (G.M.); rmellado@clinic.cat (R.M.-A.); 6 Radiology Department, Angioradiology Section, Hospital Clinic of Barcelona, 08036 Barcelona, Spain; lroman1@clinic.cat

**Keywords:** subarachnoid hemorrhage, SEBES, GCS, delayed cerebral ischemia, vasospasm

## Abstract

Background: The level of consciousness and cerebral edema are among the indicators that best define the intensity of early brain injury following aneurysmal subarachnoid hemorrhage (aSAH). Although these indicators are usually altered in patients with a poor neurological status, their usefulness for selecting patients at risk of cerebral infarction (CI) is not well established. Furthermore, little is known about the evolution of these indicators during the first week of post-ictal events. Our study focused on describing the association of the longitudinal course of these predictors with CI occurrence in patients with severe aSAH. Methods: Out of 265 aSAH patients admitted consecutively to the same institution, 80 patients with initial poor neurological status (WFNS 4–5) were retrospectively identified. After excluding 25 patients with early mortality, a total of 47 patients who underwent early CT (<3 days) and late CT (<7 days) acquisitions were included in the study. Early cerebral edema and delayed cerebral edema were calculated using the SEBES score, and the level of consciousness was recorded daily during the first week using the Glasgow Coma Scale (GCS). Results: There was a significant improvement in the SEBES (Early-SEBES median (IQR) = 3 (2–4) versus Delayed-SEBES = 2 (1–3); *p* = 0.001) and in GCS scores (B = 0.32; 95% CI 0.15–0.49; *p* = 0.001) during the first week. When comparing the ROC curves of Delayed-SEBES vs Early-SEBES as predictors of CI, no significant differences were found (Early-SEBES Area Under the Curve: 0.65; Delayed-SEBES: 0.62; *p* = 0.17). Additionally, no differences were observed in the relationship between the improvement in the GCS across the first week and the occurrence of CI (*p* = 0.536). Conclusions: Edema and consciousness level improvement did not seem to be associated with the occurrence of CI in a surviving cohort of patients with severe aSAH. Our results suggest that intensive monitoring should not be reduced in patients with a poor neurological status regardless of an improvement in cerebral edema and level of consciousness during the first week after bleeding.

## 1. Introduction

Between 10 and 30% of patients who suffer from an aneurysmal subarachnoid hemorrhage (aSAH) will develop cerebral infarctions (CIs) that are unrelated to aneurysm exclusion procedures. Infarctions that occur during the so-called period of cerebral vasospasm (between 3 and 14 days after bleeding) represent one of the main causes of disability and permanent sequelae in aSAH [1]. The limited knowledge of the pathophysiology of this complication is one of the main drawbacks in the search for reliable CI predictors. A recent hypothesis predicted that the initial inflammatory response―which occurs due to aneurysmal rupture―could be the cause [2], as this phenomenon seems to have a direct relationship with the intensity of initial damage during bleeding, which results in so-called early brain injury (EBI). Therefore, EBI could be a precursor of the inflammatory cascade and blood–brain barrier disruption that would lead to endothelial dysfunction and, finally, delayed infarction [3].

Clinical and radiological indicators of the initial inflammatory response—including cerebral edema and consciousness level impairment—have been used to measure the intensity of EBI. These early indicators seem to be related to the subsequent occurrence of delayed CI and long-term functional prognosis [2,4]. The recently introduced SEBES scale allows early cerebral edema to be quantified simply using an ordinal scale [5,6]. This score has been tested in patients with aSAH and has been related to functional prognosis and the occurrence of CI. However, in patients with a poor initial clinical status such as those with a World Federation Neurosurgical Score (WFNS) of 4 or 5, these indicators may lose their discriminative usefulness mainly because both cerebral edema and a low level of consciousness are common conditions in most of these patients at the time of hospital admission [3]. The behavior of these predictors in the days following aneurysm rupture has been poorly studied. We hypothesized that the evolution of these initial clinical and radiological biomarkers during the first week could be an efficient indicator of EBI intensity and, therefore, could represent good predictors of the subsequent occurrence of CI.

Considering all of the above information, the goal of the present study was to evaluate the association between the resolution of cerebral edema and improvement in the level of consciousness within the first-week post-aSAH as surrogate markers of inflammation and CI occurrence in patients with an initial poor neurological status at disease onset.

## 2. Materials and Methods

### 2.1. Patients

Data were collected from patients with aSAH (*n* = 265) registered consecutively in the prospective database of our institution from January 2013 to January 2018. A total of 80 patients (30%; 80/265) were admitted with a poor initial neurological status (WFNS: 4–5). Exclusion criteria for this analysis were death before the end of the cerebral vasospasm period, defined as the first two weeks after bleeding, and the presence of infarcts secondary to aneurysm treatment. Finally, 47 patients were included in the study. All of them had a computed tomography (CT) scan within the first three days after the aSAH and a second CT scan between the third and seventh day after the aSAH.

### 2.2. Classification of Edema and CI by CT Scan

The calculation of cerebral edema was performed retrospectively using the SEBES score (five categories: from 0 to 4) proposed by Ahn et al. [7]. As previously described, this scale was applied by assigning one point for the absence of visible sulci, either because of sulcal effacement or loss of gray-white matter differentiation at two predetermined levels in each hemisphere: (1) at the level of the insular cortex and (2) at the level of the centrum semiovale.

In our CT image clinical protocol, in addition to the diagnostic CT (CT0), a post-treatment or early CT (CT1) was performed within the first three days after bleeding and a follow-up or late CT (CT2) was performed between the fourth and seventh day after the SAH. If a patient had additional CT acquisitions done during the first week, the CT performed immediately after CT0 was chosen as CT1, and the later CT within the predefined time window was the follow-up (CT2). We recorded the days on which the CT scans were obtained in reference to the onset of the aSAH. The early-SEBES score was calculated in CT1 and Delayed-SEBES score was calculated in CT2. Each score was assessed twice by one investigator in two sessions separated by 6 months. In an intra-rater reliability analysis, Early and Delayed-SEBES scores showed excellent agreement on reassessment (Kappa 0.82 and 0.84, respectively; *p* < 0.001). In cases with discordance, a consensus was reached with a second investigator before the closure of the database.

The occurrence and timing of CI were prospectively registered in our database through regular multidisciplinary meetings. For this analysis, CI were defined as those occurring between the 3rd and 21st-day post-aSAH that were not related to aneurysm exclusion procedures (the infarction was not present after 24–48 h of treatment). CT hypodensities related to monitoring catheters, ventricular catheters, or intraparenchymal hematomas were not considered as CI for the purpose of this study.

### 2.3. Neurological Evaluation

A daily neurological evaluation of all patients was performed during the first week using the GCS. In cases in which the patients were under sedoanalgesia without any motor or verbal responses, the GCS score was recorded as 3. Whenever possible, all patients had their sedation/paralysis interrupted/reversed for the GCS assessment.

### 2.4. Statistical Analysis

Continuous variables were reported as the mean (standard deviation) or median (interquartile range) values and were compared using the Student *t*-test, Mann–Whitney *U* test, or Kruskal–Wallis test, as appropriate. Categorical variables were compared using the chi-square and Fisher’s exact tests. The inter-rater agreement was evaluated with Cohen’s Kappa statistic by reanalyzing the SEBES score at 6 months. A receiver operating characteristic (ROC) curve analysis was performed to assess the accuracy of the SEBES scale for predicting the occurrence of CI. To assess the improvement of edema, we constructed a Kaplan–Meier curve where the SEBES score was considered to be an ordinal variable divided into three categories, namely high-grade (3–4), low-grade (1–2), and no edema (0). Edema improvement (censored) was considered to be a shift from a superior group to an inferior one among the curve of serial CT images (CT0–CT2). The Wilcoxon (Breslow) test was used to compare survival curves. The analysis of the longitudinal course of improvement in the level of consciousness (GCS) with respect to days of admission was done using a linear regression. An additional logistic regression model was built to identify individual factors related to the delayed-SEBES score. The day of hospital admission was registered as day 0 (first 24 h). In the graphical representation of this prediction, the mean and 95% confidence intervals are presented. All statistical analyses were performed using the Stata/IC version 14.0 software for Mac, StataCorp, College Station, TX, USA, 2015.

## 3. Results

From the 80 initially-screened poor-grade aSAH patients, a total of 25 patients (25/80; 31%) died during the first two weeks of their aSAH. Among the survivors, eight (10%) had significant infarctions secondary to aneurysm treatment during the first week and were also excluded from the study. Finally, 47 patients (59%; 47/80) who survived the first two weeks and had early and delayed brain CT acquisitions were included in the study. Regarding the types of treatments experienced by these patients, 7 (15%; 7/47) underwent surgical treatment, and 40 (85%; 40/47) had endovascular treatment. The anterior communicating artery (ACoA) was the most frequent aneurysm location (14 patients; 30%; 14/47), followed by the posterior communicating artery (PCoA; 8 patients; 17%; 8/47), the internal carotid artery (ICA; 7 patients; 15%; 7/14) and the middle cerebral artery (MCA; 5 patients; 11%; 5/47). Finally, 7 patients (15%; 7/47) exhibited aneurysms in other locations of the posterior vascular territory, and 6 (13%; 6/47) had aneurysms in other locations of the anterior vascular territory. With respect to the initial amount of blood, according to the modified Fisher scale used to evaluate the admission CT scans, 44 patients (94%; 44/47) had a score of 4, 2 patients (4%; 2/47) had a score of 2, and only one patient (2%; 1/47) had a score of 1.

In this cohort, a total of 11 patients (23%; 11/47) developed CI (Table 1). In the univariate analysis, the only variable found to be significantly associated with the occurrence of CI was sex (16% for women versus 43% for men, *p* = 0.04). The rest of the variables included in the analysis, such as smoking, hypertension, diabetes mellitus, and hydrocephalus were not significantly related to subsequent CI occurrence. A logistic regression showed that high-grade delayed SEBES was not associated with individual factors such as age (OR: 0.96; 95% CI, 0.92–1.01) and sex (OR: 0.36; 95% CI, 0.09–1.45).

**Figure 1:**
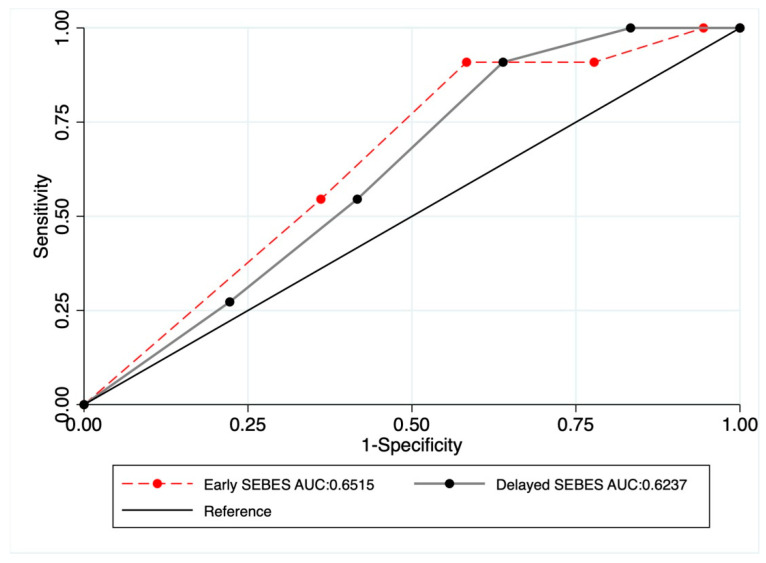
Receiver operating characteristics (ROC) and area-under-the-curve (AUC) analyses for SEBES (Early and Delayed SEBES) and the occurrence of Cerebral Infarctions (CI).

The distribution of the SEBES scores obtained in the early CT (<3 days, Early-SEBES) and late CT (3–7 days, Delayed-SEBES) acquisitions, according to the occurrence of CI, is shown in Table 1. Of note, there was a reduction in the SEBES score of one point between the early CT and late CT evaluations (median (IQR) 3 (2–4) versus 2 (1–3) respectively, *p* = 0.001). As shown in Figure 1, the prognostic value of the SEBES scores did not change across the two time points (Early-SEBES, OR = 1.55; 95% CI, 0.32–2.88; Delayed-SEBES, OR = 1.45;95% CI, 0.83–2.52), and there were no differences between the ROC curves (Early-SEBES area under the curve (AUC) = 0.65; 95% CI, 0.49–0.81 versus Delayed-SEBES AUC = 0.624; 95% CI, 0.46–0.79; *p* = 0.17).

**Figure 2:**
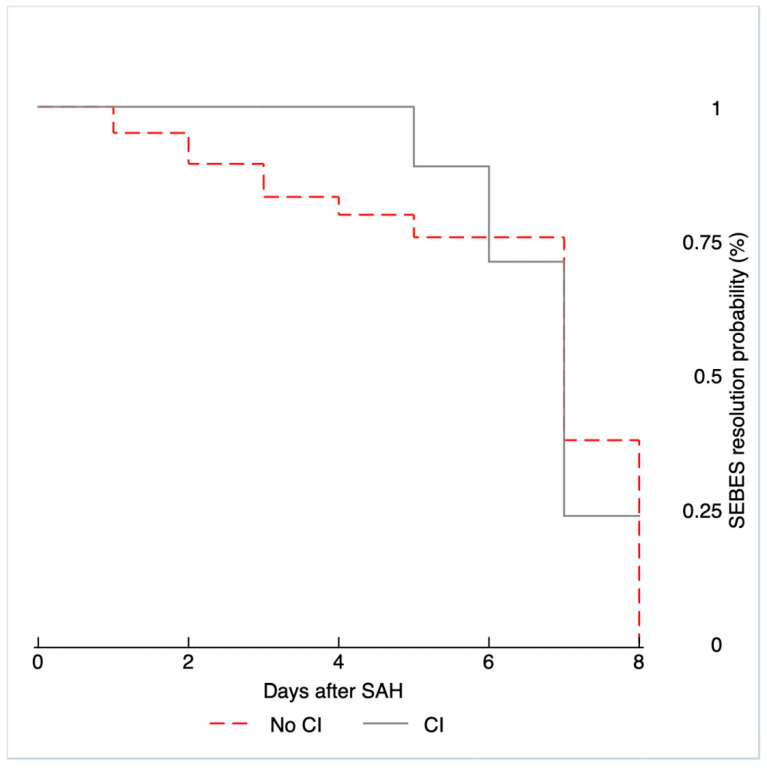
Kaplan–Meier curves showing cerebral edema improvement for CI and no-CI patients during the first week.

The Kaplan–Meier curve depicted in Figure 2 represents the evolution of edema including every CT scan performed on each patient during the first week. The curve shows the time point at which each patient changed from belonging to the high edema group (SEBES 3–4) to a lower edema group. As illustrated, no difference in the continuous edema resolution within the group of patients who experienced CI and those who did not was observed *(p =* 0.398).

**Figure 3:**
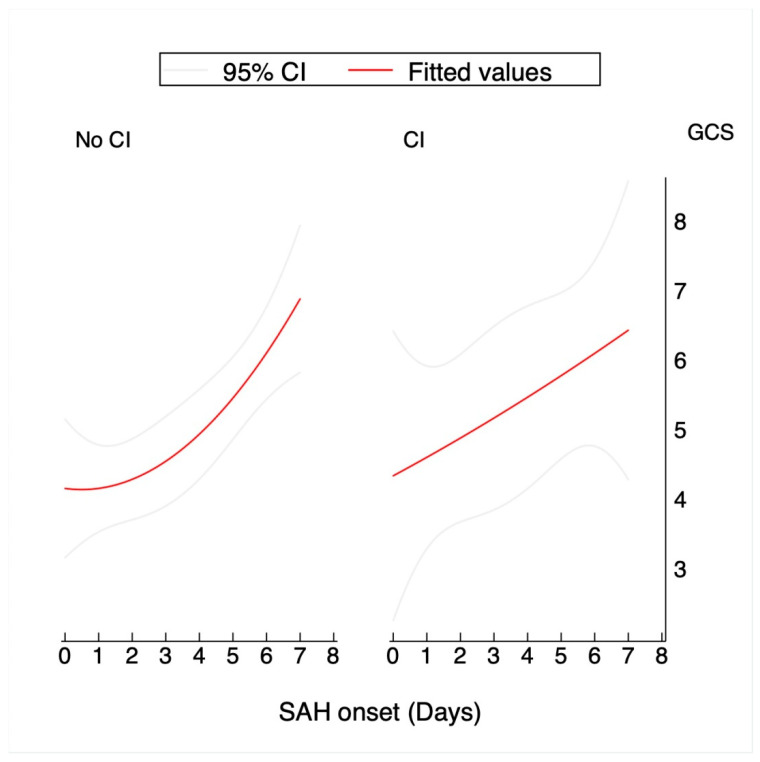
Graphic illustration of the prediction of the mean Glasgow Coma Scale (GCS) (read lines) during the first week: a comparison of CI and non-CI patients. The gray lines represent the confidence interval (95%) of the predicted mean.

Regarding the longitudinal neurological evaluations performed during the first week, there was a significant improvement in the level of consciousness measured daily through the GCS across the first week after bleeding (B = 0.36; 95% CI, 0.19–0.53); *p* = 0.001. The longitudinal improvement trajectories for the GCS score did not differ between the CI and no-CI groups in the linear regression model (*p* = 0.536), as shown in Figure 3.

## 4. Discussion

In aSAH patients, EBI resulting from the initial bleed is believed to be a precursor to the pathophysiological cascade that could ultimately result in delayed infarction [8]. Prior work has documented the effectiveness of cerebral edema and initial neurological assessment as good predictors of delayed infarcts; thus, they are considered to be good biomarkers of EBI [2]. However, their value through the first week of aSAH evolution in poor-grade aSAH patients is unknown. The perpetuation of brain damage―determined by SEBES and daily GCS―can be decisive for CI occurrence. Here, we aimed to describe the association between the temporal course of these main EBI indicators within the first week after aSAH and the occurrence of CI. We selected these predictors due to their importance in the definition of EBI and the lack of studies in the literature addressing their changes during the subacute phase [2]. In addition, we focused on a cohort of severe patients in which EBI is frequent; thus not allowing to initially pick out those individuals with a higher risk of CI.

### 4.1. EBI in Severe aSAH Patients

Mortality and poor functional prognosis of patients with aSAH and poor initial neurological status are currently very significant problems. In accordance with previous studies, our cohort showed a mortality rate of 41% during the first week after aSAH [9]. However, despite the severity of the disease, about one-third of the survivors reaches a good functional outcome after three months [10,11,12]. Thus, there is a subgroup of patients that seems to survive with an acceptable functional prognosis despite their initial poor clinical condition. Determining the phenomena related to good clinical prognosis in these survivors should be a key aspect in determining therapeutic efforts [13]. In these patients, the long-term functional status seems to depend largely on the occurrence of EBI, and the EBI intensity is believed to be directly related to CI occurrence [8]. Nevertheless, in these patients with poor initial neurological status, our proposed indicators of EBI are quite common and thus may lose their prediction value.

### 4.2. Biomarkers of EBI and Predictors of CI

There is a lack of reliable biomarkers that can predict CI occurrence in the early phase of aSAH evolution. A few laboratory studies have related certain blood biomarkers to the occurrence of CI, but those biomarkers were assessed in a delayed fashion after the first days of brain damage [14,15,16,17]. Some authors have also related delayed clinico-radiological indicators (after the third day) to functional prognosis, yet those biomarkers were not found to be directly associated with the occurrence of CI. Rass et al. recently observed how a decrease in cerebral edema in a cohort of severe aSAH patients was associated with a better functional prognosis [5]. However, they could not reproduce this association with the occurrence of CI. Studies assessing delayed perfusion CTs have also related certain perfusion defects with a worse functional prognosis, although those perfusion defects did not seem to relate to CI occurrence in patients with severe aSAH [18]. The research effort of this work was focused on studying the resolution of EBI in the first week after the occurrence of aSAH as it could serve as an early predictor of CI and might be a promising new target.

### 4.3. SEBES Scale and GCS During the First Week

To investigate EBI resolution, our study assessed the relationship between two clinico-radiological predictors: edema and clinical evolution during the first week with subsequent CI occurrence. In our cohort of poor-grade aSAH survivors, we observed a significant improvement in cerebral edema during the first week. This finding is in line with other studies, which have indicated a gradual resolution of edema during the first days after aSAH [5]. Our data showed that both Early-SEBES and Delayed-SEBES seemed to have a similar predictive value for the risk of CI occurrence in poor-grade aSAH patients. Indeed, a comparison of the ROC curves of early and late edema did not show significant differences in the prediction of the subsequent appearance of CI. Accordingly, a greater resolution of edema did not seem to imply a lower probability of CI occurrence in patients with severe aSAH.

The initial level of consciousness continues to be one of the most robust predictors of subsequent CI occurrence [3,19]. However, its value as a predictor of CI during the first week in patients with severe aSAH is unknown. An improvement in the level of consciousness measured by the GCS daily could also be a good indicator of EBI, as it is an indirect measure of inflammation persistence that would increase the probability of CI occurrence. Focusing on this variable during the first week, we observed significant clinical improvement of the survivors (B = 0.36; *p* = 0.001). However, and similarly to what happens with edema, the improvement in the neurological evaluations was not related to a lower probability of CI occurrence.

### 4.4. Other Factors Influencing EBI Resolution

It should also be considered that the persistence of the inflammatory response may differ depending on a patient’s initial characteristics. Age, for example, seems to be related to the greater occurrence of CI and a worse functional prognosis [11]. The role of gender remains a matter of debate, as data on its relationship with CI incidence varies widely from one study to another, likely based on differences in the selected cohorts and the definitions of ischemia or CI [20,21]. Our study showed an association between female gender and a lower CI incidence. However, neither age nor gender were found to be related to delayed SEBES. Therefore, age would be related to a greater occurrence of CI but seems to have a neutral or negative correlation with the delayed SEBES score [11]. Arguably, a higher percentage of brain atrophy in older patients would explain the lower level of cerebral edema, thus accounting for our findings. Other indicators such as smoking, hypertension, diabetes mellitus, and hydrocephalus were not found to be significantly related to subsequent CI occurrence in our univariate analysis, which is in line with the results of previous studies that have not shown strong relationships of these factors with CI incidence [18,21,22,23].

### 4.5. Novel Findings

There is increasing evidence that EBI could be a trigger for the subsequent occurrence of CI and that the inflammatory response has a relevant role in secondary damage [11,24]. In the present study, we focused on the value of two widely-used clinico-radiological indicators of EBI measured during the first week after aSAH occurrence. In this surviving cohort of patients with severe aSAH, the temporal course of the SEBES and GCS scores was not shown to be an effective predictor of CI. These observations argue against reducing the close monitoring of patients who exhibit improvements in edema or level of consciousness and should be taken into account in therapeutic decision-making for severe patients during the subacute phase of aSAH evolution. Further research effort should focus on the identification of new laboratory biomarkers or advanced monitoring techniques for the early diagnosis of CI.

### 4.6. Limitations

Our work has limitations worth mentioning. Although we started with a significant total number of patients, the final cohort included in the study was limited since there was a significant loss of patients due to the exclusion criteria. However, the use of these demanding exclusion criteria allowed us to focus on the survivors, as it was relevant to detect those patients who exhibited good clinical evolution over a long-term period. Another conflicting aspect might be the definition of secondary injury, which varies among different publications. In the present study, we defined CI as a secondary infarct detected between the 3rd and 21st days of aSAH onset that was not related to aneurysmal exclusion procedures. It is worth mentioning that some of these CI detected by CT scans may not have had significant implications on functional prognosis.

## 5. Conclusions

In patients with poor-grade aSAH, cerebral edema resolution and neurological recovery did not seem to have a strong impact on the prediction of CI occurrence. Although a reduction in edema and improvement in GCS values were observed during the first week after aSAH, the temporal changes in these indicators within the first week after aSAH were not informative of CI occurrence. These findings should be taken into account in the interpretation of delayed CT and the implementation of management decisions for poor-grade aSAH patients.

## Figures and Tables

**Table 1:** Demographic of DCI (Delayed Cerebral Infarctions) risk factors of patients included in the study.

	CI *n* = 11	No CI *n* = 36	*p*
mRS (0–3)	4 (36.36%)	15 (41.67%)	0.754
Age	54.27(SD:12.47)	60.81 (SD:12.85)	0.140
Gender (female)	5 (45.45%)	28 (77.78%)	0.040
HBP	4 (36.36%)	16 (44.44%)	0.635
Smoke	5 (45.45%)	12 (33.33%)	0.464
DM	2 (18.18%)	2 (5.56%)	0.189
DLP	5 (45.45%)	13 (36.11%)	0.577
Hydrocephalus	9 (81.82%)	29 (80.56%)	0.926
ICH	5 (45.45%)	13 (36.11%)	0.577
Hijdra Sum Score	29.4 (SD:5.7)	23.9 (SD:10.7)	0.108
Vasospasm	11 (100%)	16 (44.4%)	0.001
Rescue therapy	5 (45.4%)	4 (11.1%)	0.015
Early SEBES			
0	0	2 (5.56%)	
1	1 (9.09%)	6 (16.67%)	
2	0	7 (19.44%)	
3	4 (36.36%)	8 (22.22%)	
4	6 (54.55%)	13 (36.11%)	
			0.607
Delayed SEBES			
0	0	6 (16.67%)	
1	1 (9.09%)	7 (19.44%)	
2	4 (36.36%)	8 (22.22%)	
3	3 (27.27%)	7 (19.44%)	
4	3 (27.27%)	8 (22.22%)	
			0.500
GCS (day)			
0	6.2 (SD:1.29)	5.48 (SD:0.59)	
1	3.7 (SD:0.7%)	3.26 (SD:0.26)	
2	4.9 (SD:1.32)	3.84 (SD:0.49)	
3	5 (SD:1.31)	4.39 (SD:0.65)	
4	6.1 (SD:1.64)	5.1 (SD:0.71)	
5	6.1 (SD:1.63)	5.64 (SD:0.75)	
6	6.1 (SD:1.63)	6.32 (SD:0.79)	
7	6.1 (SD:1.63)	6.35 (SD:0.8)	

mRS: Modified Rankin Scale; HBP: High blood pressure; DM: Diabetes Mellitus; DLP: Dyslipidemia; ICH: Intracranial Hemorrhage. Vasospasm: Angiographic Vasospasm diagnosed by either CT-angio scan or arteriography; Rescue therapy: Endovascular rescue therapy with intra-arterial administration of verapamil.

## Data Availability

The data presented in this study are available on request from the corresponding author. The data are not publicly available due to containing information that could compromise the privacy of research participants.
